# Endofin, a novel BMP-SMAD regulator of the iron-regulatory hormone, hepcidin

**DOI:** 10.1038/srep13986

**Published:** 2015-09-11

**Authors:** Justin B. Goh, Daniel F. Wallace, Wanjin Hong, V. Nathan Subramaniam

**Affiliations:** 1 QIMR Berghofer Medical Research Institute, Brisbane, Australia; 2 Faculty of Medicine and Biomedical Sciences, The University of Queensland, Brisbane, Australia; 3 Institute of Molecular and Cell Biology, Singapore

## Abstract

BMP-SMAD signalling plays a crucial role in numerous biological processes including embryonic development and iron homeostasis. Dysregulation of the iron-regulatory hormone hepcidin is associated with many clinical iron-related disorders. We hypothesised that molecules which mediate BMP-SMAD signalling play important roles in the regulation of iron homeostasis and variants in these proteins may be potential genetic modifiers of iron-related diseases. We examined the role of endofin, a SMAD anchor, and show that knockdown of endofin in liver cells inhibits basal and BMP-induced hepcidin expression along with other BMP-regulated genes, *ID1* and *SMAD7*. We show for the first time, the *in situ* interaction of endofin with SMAD proteins and significantly reduced SMAD phosphorylation with endofin knockdown, suggesting that endofin modulates hepcidin through BMP-SMAD signalling. Characterisation of naturally occurring SNPs show that mutations in the conserved FYVE domain result in mislocalisation of endofin, potentially affecting downstream signalling and modulating hepcidin expression. In conclusion, we have identified a hitherto unrecognised link, endofin, between the BMP-SMAD signalling pathway, and the regulation of hepcidin expression and iron homeostasis. This study further defines the molecular network involved in iron regulation and provides potential targets for the treatment of iron-related disorders.

The transforming growth factor-β (TGFβ) family is composed of 33 structurally related growth factors including TGFβ and bone morphogenetic proteins (BMPs) which regulate a wide variety of cellular responses such as proliferation, differentiation and apoptosis^1^,^2^,^3^,^4^. The TGFβ family members bind to two distinct serine/threonine kinase receptors on cellular membranes, termed type I and type II receptors^5^,^6^. Upon ligand binding, type II receptors phosphorylate type I receptors at the glycine-serine (GS) domain resulting in phosphorylation and activation of receptor-regulated small mothers against decapentaplegic (R-SMADs)^7^,^8^. TGFβ and activin signals activate the R-SMADs: SMAD2 and SMAD3, while BMP signals activate SMAD1, SMAD5, and SMAD8^9^,^10^. R-SMADs subsequently form a heterocomplex with the common mediator SMAD, SMAD4, before translocating to the nucleus to activate the transcription of various target genes^11^.

SMAD anchors are recently identified adaptor molecules which facilitate interactions between TGFβ receptors and SMAD proteins, enhancing signal transduction and expression of target genes^12^,^13^. TGFβ signalling is mediated by the SMAD anchor for receptor activation (SARA) while signalling in the BMP pathway is mediated by the endosome-associated FYVE-domain protein (endofin)^14^. Endofin, encoded by the *ZFYVE16* gene, contains a conserved double zinc finger, the FYVE (Fab-1, YGL023, Vps27, and EEA1) domain^14^,^15^. The FYVE domain is a cysteine-rich Zn^2+^ binding domain with a basic motif that binds to phosphatidylinositol-3-phosphate (PI3P), a lipid highly enriched in early endosomes^16^,^17^. Therefore, the FYVE domain is consequently responsible for localising endofin to endosomal vesicles which is important for its function in trafficking SMADs to internalised BMP receptors in early endosomes^14^. Perturbing the FYVE-domain, as with the previously reported C753S mutation, mislocalises endofin and attenuates BMP signalling^13^,^14^.

Disruption of TGFβ superfamily signalling has been associated with cancer, cardiovascular, fibrotic and skeletal diseases^18^. BMP-SMAD signalling is also central to iron homeostasis through the regulation of hepcidin, a peptide hormone encoded by the *HAMP* gene, responsible for regulating serum iron levels. Most iron disorders are associated with a dysregulation of hepcidin. Iron overload resulting from hepcidin deficiency causes hyperabsorption of dietary iron and can lead to tissue and organ damage^19^. In contrast, excess hepcidin limits dietary iron absorption and macrophage iron recycling, restricting iron availability to erythrocyte precursors and eventually causing iron-restricted anaemia. As such, hepcidin regulation is tightly controlled at many levels. This includes BMPs which bind to trans-membrane BMP receptors, facilitated by hemojuvelin (HJV), a co-receptor that enhances BMP signalling to induce the transcription of *HAMP*, together with other BMP-regulated genes such as *ID1* (inhibitor of DNA binding protein 1) and *SMAD7* (SMAD family member 7)^20^,^21^,^22^.

We hypothesised that endofin plays an important role in the regulation of hepcidin as it has been shown to be important in BMP-SMAD signalling. In this study, we investigated the potential role of endofin in hepcidin regulation in the BMP-SMAD pathway. In addition, we characterised naturally-occurring SNPs affecting the conserved FYVE domain of endofin to elucidate potential effects of endofin polymorphisms on BMP signalling and hepcidin regulation. Our finding further defines the molecular network involved in iron regulation, providing potential targets for the treatment of iron-related disorders and possibly BMP-associated disorders.

## Results

### Endofin knockdown down-regulates basal hepcidin expression

**Figure 1:**
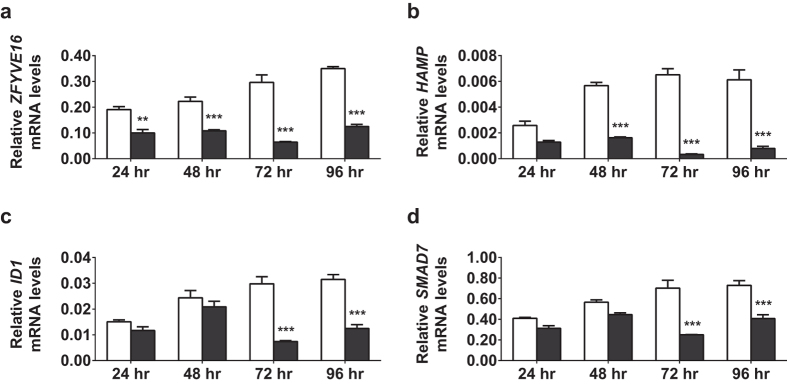
Endofin silencing is associated with reduced basal hepcidin expression. HepG2/C3A cells were transfected with either non-specific siRNA (si-NS, white bars) or endofin siRNA (si-Endofin, black bars) from 24 to 96 hr. The mRNA expression of (**a**) *ZFYVE16* (Endofin), (**b**) *HAMP*, (**c**) *ID1* and (**d**) *SMAD7* were measured by qPCR and normalised to the geometric means of the reference genes *ACTB* and *HPRT*. Data are representative of three independent biological experiments. Bars represent means ± standard errors of triplicates. *P*-values of endofin knockdown relative to si-NS controls were calculated using 2-way ANOVA; *indicates a *p*-value of < 0.05, **p < 0.01, ***p < 0.001.

To determine the role of endofin on hepcidin regulation, we first examined the effect of modulating endofin levels by treating the human hepatic cell line, HepG2/C3A, with either non-specific siRNA or endofin-specific siRNA. Cells were incubated with siRNAs from 24 hr to 96 hr. We observed that endofin expression, as measured by quantitative real time PCR (qPCR), was significantly decreased compared to controls (Fig. 1a). Endofin expression was significantly decreased at all time points, being at approximately 50% of control levels at 24 hr (p = 0.006), with a maximum of 70% knockdown at 72 hr (p < 0.001). Endofin expression remained effectively decreased up to 96 hr post transfection.

Following endofin knockdown, we observed a significant decrease in basal *HAMP* expression in C3A cells across all time points (Fig. 1b). Similar to endofin, *HAMP* expression was maximally reduced at 72 hr (p < 0.001), down to 10% of control levels and remained decreased at 96 hr (p < 0.001).

Analysis of BMP-regulated genes, *ID1* and *SMAD7*, containing BMP responsive elements in their promoters, showed that basal levels were decreased following endofin knockdown, although the amount of decrease was less than that of *HAMP* and only statistically significant at the 72 hr (p < 0.001) and 96 hr time points (p < 0.001) (Fig. 1c,d).

### BMP6 induction of hepcidin is disrupted by endofin knockdown

**Figure 2:**
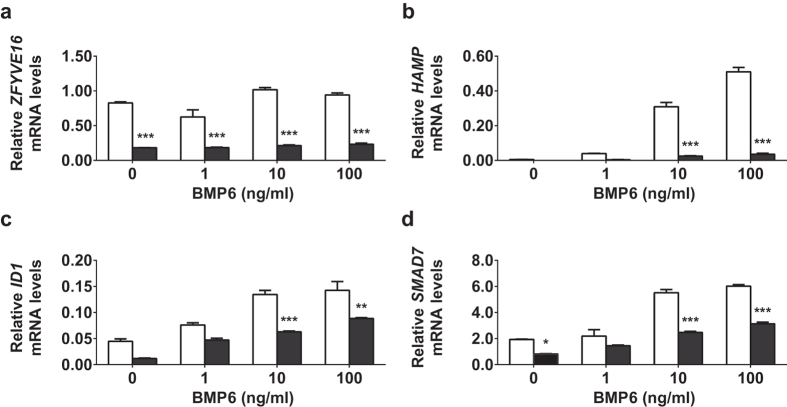
BMP-induced *HAMP*, *ID1* and *SMAD7* levels are decreased following endofin knockdown. C3A cells were transfected with either non-specific siRNA (si-NS, white bars) or endofin siRNA (si-*Endofin*, black bars) for 72 hr and treated with BMP6 at 1, 10 or 100 ng/ml for 4 hr. The mRNA expression of (**a**) *ZFYVE16* (Endofin), (**b**) *HAMP*, (**c**) *ID1* and (**d**) *SMAD7* were measured by qPCR and normalised to the geometric means of the reference genes *ACTB* and *HPRT*. Data are representative of three independent biological experiments. Bars represent means ± standard errors of triplicates. *P*-values were calculated using 2-way ANOVA; *indicates a *p*-value of < 0.05, **p < 0.01, ***p < 0.001.

We next analysed the ability of BMP6 to regulate hepcidin in the absence of endofin. C3A cells were incubated with control and endofin-specific siRNA for 72 hr and subsequently treated with BMP6. Increasing amounts of BMP6 treatment at concentrations of 1, 10 or 100 ng/ml for 4 hr increased *HAMP* expression by approximately 10-, 60- and 100-fold respectively. Endofin expression was significantly decreased in cells treated with endofin-specific siRNA, regardless of BMP6-treatments (p < 0.001) (Fig. 2a). Despite BMP6-induction of *HAMP* at all concentrations, we observed up to 90% decreased expression following endofin silencing (Fig. 2b). This decrease in *HAMP* expression following endofin knockdown was similar to that observed in untreated C3A cells, expressing at approximately 10% of non-specific siRNA control levels.

BMP-regulated genes *ID1* and *SMAD7* were also inhibited following endofin knockdown and BMP6 treatment. Although, the magnitude of induction by BMP6 and inhibition following endofin knockdown was less than observed with *HAMP* expression (Fig. 2c,d).

### Duolink Proximity Ligation Assay shows *in situ* interaction of SMAD1 with endofin but not SARA

To assess if endofin modulates hepcidin through the BMP-SMAD signalling pathway, we analysed SMAD1 interactions with endofin using a proximity ligation assay (PLA). PLA is a new technology which allows for *in situ* detection of protein-protein interactions. PLA utilises antibody recognition coupled with PLA probes which generate a localised signal in a form of spots, revealing proteins which are in close proximity (<40 nm). In this assay, we used as controls the SMAD anchor SARA and SMAD2/3, which are closely related signalling components of the TGFβ but not the BMP pathway. C3A cells were transfected with either myc-tagged endofin or myc-tagged SARA constructs for 48 hr before processing for PLA.

**Figure 3:**
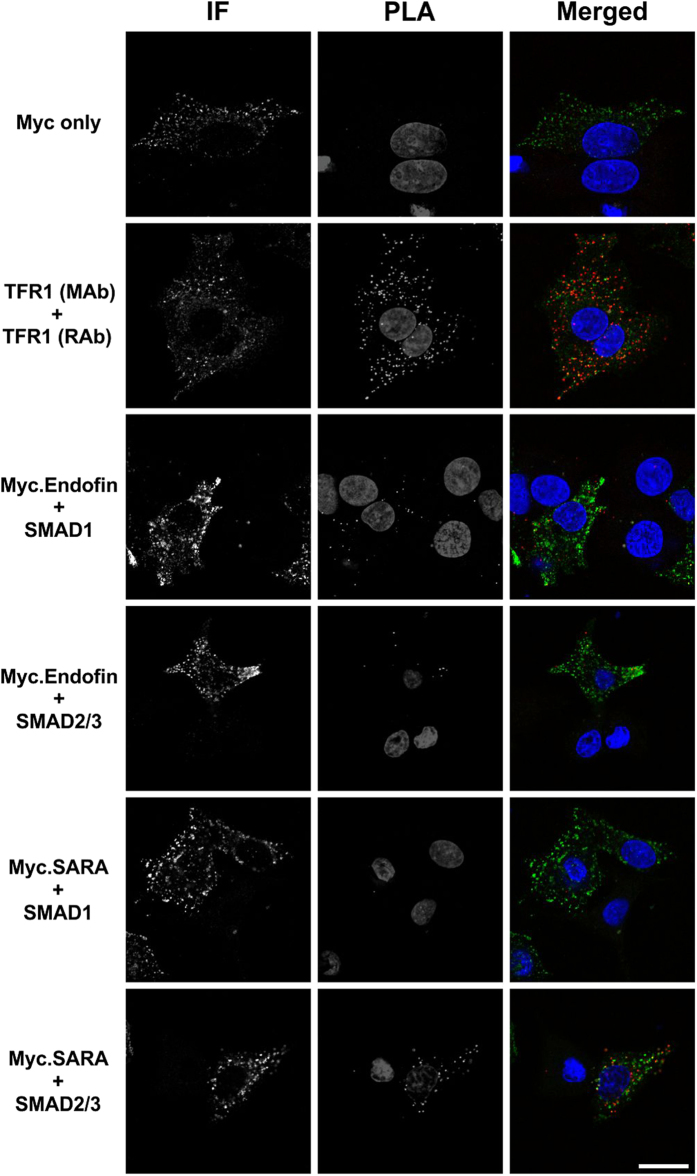
Proximity ligation assay shows that endofin but not SARA interacts with SMAD1. C3A cells were transfected with either myc-tagged endofin or myc-tagged SARA plasmids for 48 hr before processing for PLA with myc, SMAD1, and SMAD2/3 total antibodies. As a negative control, C3A cells were incubated with one antibody (top panel) and for a positive control, we used endogenous TfR1 which forms homodimers. The remaining panels show cells incubated with two antibodies to assess SMAD anchor and SMAD protein interaction. Cells were incubated with anti-mouse secondary antibodies after the PLA experiment to identify transfected cells (left panel). The middle panel shows PLA assessment of SMAD anchor and SMAD interaction and the right panel shows a merged immunofluorescence (IF) and PLA image. Nuclei were stained with DAPI as shown in the middle and right panel. Data are representative of three independent biological experiments. Scale bar represents 20 μm.

Through PLA, we observed endofin interaction with endogenous SMAD1 proteins, localised in cytoplasmic regions of the cell (Fig. 3). We found that endofin also interacted with SMAD2/3 although fewer signal spots were observed. We show that SARA interacts exclusively with SMAD2/3 and not SMAD1 (Fig. 3). These interactions were only observed in transfected cells indicating the specificity of the assay. Cells incubated with single antibodies showed low or no signal and were used to assess background signal (Fig. 3).

### SMAD1/5/8 phosphorylation is reduced with endofin knockdown

To determine if the regulation of hepcidin expression by endofin occurs through the BMP-SMAD signalling pathway, we analysed the phosphorylation of SMAD1/5/8 proteins following endofin knockdown. C3A cells were knocked-down for endofin and treated with BMP6 (10 ng/ml) for 4 hr. In addition to protein analysis, mRNA levels of *HAMP*, *ID1* and *SMAD7* were measured by qPCR to verify the downstream effects on BMP-regulated gene transcription.

**Figure 4:**
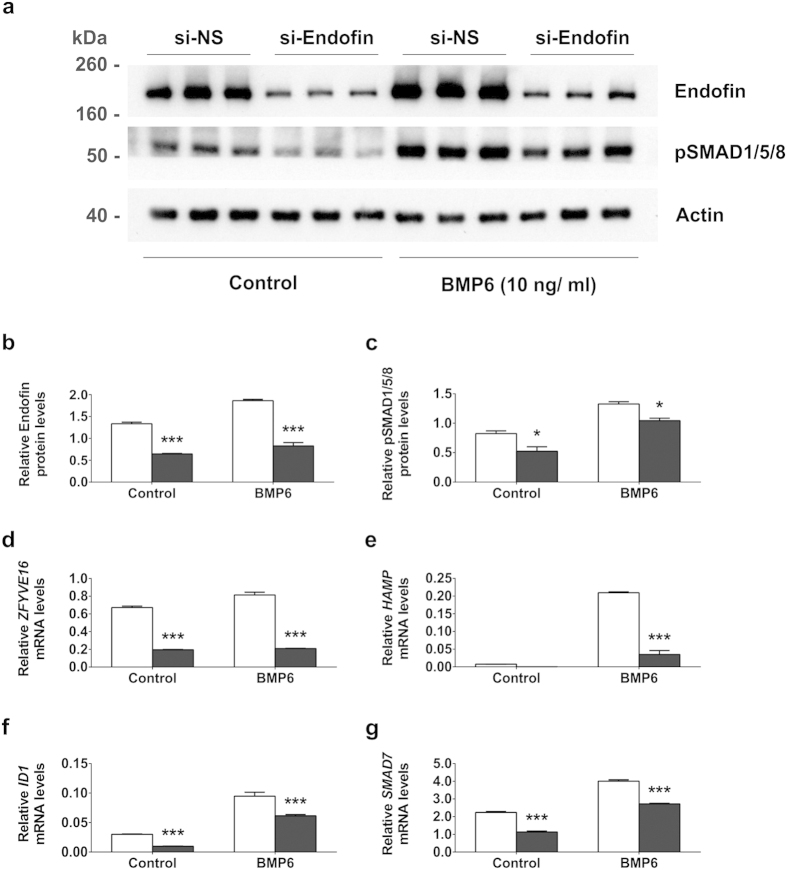
Endofin silencing reduces phosphorylation of SMAD1/5/8 proteins. C3A cells were transfected with non-specific siRNA (si-NS) or endofin-specific siRNA (si-Endofin) for 72 hr before treating with BMP6 (10 ng/ml) for 4 hr. (**a**) Cells were harvested and lysates were immunoblotted with antibodies against endofin, phospho-SMAD1/5/8. β-actin was used as a loading control. Bands developed on film were scanned and densitometry was used to quantify the levels of endofin (**b**) and pSMAD1/5/8 proteins (**c**) relative to actin using the GeneGenius Imaging System. The mRNA expression of (**d**) *ZFYVE* (Endofin), (**e**) *HAMP*, (**f**) *ID1* and (**g**) *SMAD7* were measured by qPCR and normalised to the geometric means of the reference genes *ACTB* and *HPRT*. Data are representative of three independent biological experiments. Bars represent means ± standard errors of triplicates. *P*-values of endofin knockdown relative to si-NS controls were calculated using 2-way ANOVA; *indicates a p-value of < 0.05, **p < 0.01, ***p < 0.001.

After a 72 hr treatment with endofin-specific siRNA, immunoblotting showed reduced endofin protein levels in both BMP6 treated and untreated C3A cells (Fig. 4a,b). We observed a significant increase in SMAD1/5/8 phosphorylation in cells treated with BMP6 (p < 0.001), as reported previously^23^. Knockdown of endofin resulted in significantly decreased SMAD1/5/8 phosphorylation compared to controls in both BMP6 treated (p = 0.022) and untreated C3A cells (p = 0.016) (Fig. 4c). Levels of endofin mRNA were decreased which corresponds to the decrease in endofin protein levels (Fig. 4d). Consistent with the reduction in SMAD1/5/8 phosphorylation, in the same cells, *HAMP*, *ID1* and *SMAD7* mRNA showed reduced basal and BMP-induced levels following endofin knockdown (Fig. 4e–g).

### Polymorphisms in the FYVE domain affects proper endofin localisation

**Figure 5:**
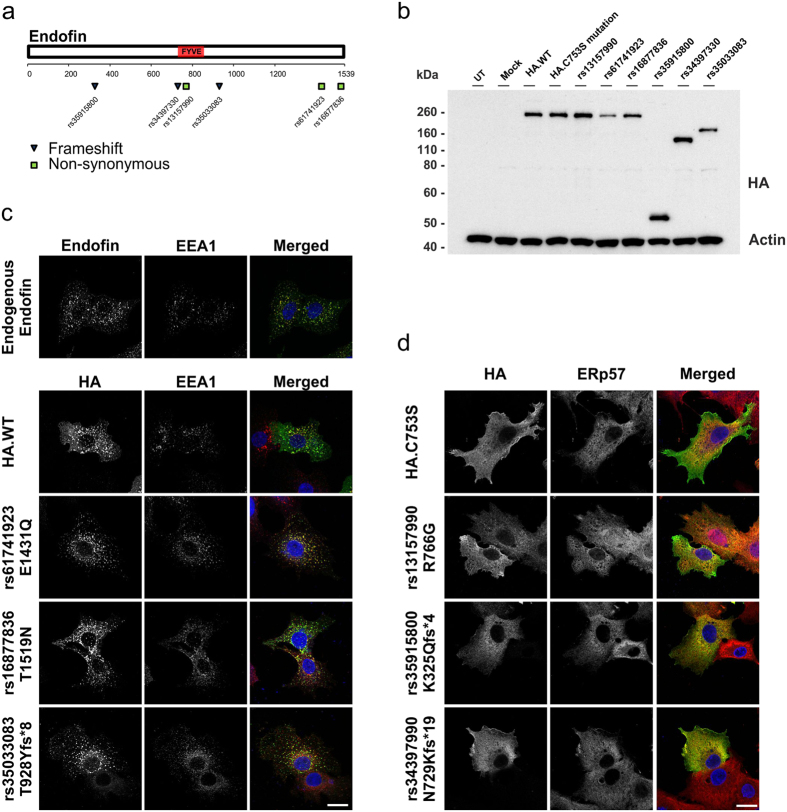
Mutations disrupting the conserved FYVE domain of endofin cause mislocalisation. Plasmid constructs containing six naturally occurring endofin SNPs identified to be potentially deleterious were generated through site-directed mutagenesis and transfected into C3A cells. After 48 hours, cells were immunostained with anti-HA antibodies (first column). Cells were also immunostained with organelle markers, EEA1 and ERp57 as represented in the second column. The nuclei were stained with DAPI as represented in the third column which is an overlay of the first and second panels. (**a**) Schematic diagram of endofin protein structure with the FYVE domain (red rectangle), and mapped non-synonymous (boxes) and frameshift (triangles) mutations. (**b**) Western blotting showing the corresponding sizes of proteins containing non-synonymous and frameshift SNPs with premature stop codons. (**c**) Panels show C3A cells transfected with endofin SNP variants without an affected FYVE domain. (**d**) Panels show C3A cells transfected with SNP variants affecting the FYVE domain. Data are representative of three independent biological experiments. Scale bar represents 20 μm.

As decreased endofin expression modulated hepcidin levels, which in turn would result in increased body iron levels, we reasoned that single nucleotide polymorphisms (SNPs) which affected endofin localisation or function might have a similar effect. We identified naturally occurring endofin SNP variants through SNP database searches and analysed using the SIFT (Sorting Intolerant From Tolerant) and PolyPhen-2 (Polymorphism Phenotyping v2) protein prediction tools to determine potentially deleterious mutations and generated them through site-directed mutagenesis of HA-epitope tagged endofin constructs. These were transiently transfected into C3A cells (Fig. 5a,b).

Intracellular endofin expression was analysed by immunofluorescence and confocal microscopy to determine the consequences of SNPs on endofin localisation. We observed endogenous endofin as vesicle-like structures in the cytoplasm that resembled endosomes. Co-localisation studies with an endosomal marker, early endosome antigen 1 (EEA1), showed a degree of overlap (Fig. 5c). Transfection with wild-type HA-epitope tagged endofin constructs showed similar localisation patterns with some vesicles noted to be slightly enlarged as compared to endogenous endofin. Analysis of three endofin variants (E1431Q, T1519N and T928Yfs*8) all possessing an intact FYVE domain showed endosomal-like localisation patterns, similar to wild-type constructs. However, one non-synonymous (R766G) and two frameshift mutations (K325Qfs*4 and N729Kfs*19) with disrupted FYVE domain regions showed a diffused cytoplasmic localisation pattern. Co-localisation with the ER marker, ERp57, showed a degree of overlap (Fig. 5d). A similar ER localisation was also observed with the HA-tagged endofin-C753S mutant that also has a disrupted FYVE domain and has been shown previously to lead to incorrect localisation of the protein^14^,^24^.

## Discussion

Previous studies have shown that endofin enhances BMP-SMAD signalling by anchoring SMAD proteins to BMP receptors. Here we have identified endofin as a novel signalling component required for hepcidin regulation, by mediating SMAD1/5/8 phosphorylation in the BMP-SMAD pathway. We also show that variants which affect endofin localisation may have an effect on hepcidin expression and could potentially play roles as genetic modifiers of iron overload.

Knockdown of endofin with specific siRNA results in down-regulation of basal hepcidin expression. Knockdown of endofin also leads to a down-regulation of BMP-regulated genes, *ID1* and *SMAD7*, indicating that endofin is essential for the basal expression of genes in the BMP-SMAD pathway. Our results support a previous study where a mutant and non-functional form of endofin, the FYVE (C753S) mutant, transfected in mouse myoblast C2C12 cells was found to decrease expression of a BMP-responsive reporter gene^13^. Conversely, stable constructs of the wild-type form slightly enhanced reporter gene expression.

Several BMPs have been shown to induce hepcidin expression *in vitro*^25^,^26^,^27^. Only BMP6 was found to be an essential and non-redundant regulator of hepcidin and iron *in vivo*^25^. To determine if endofin regulates BMP-induced hepcidin expression, we silenced endofin and examined hepcidin expression in BMP6-treated C3A cells. Despite 100-fold induction of hepcidin with BMP6, we observed an inhibition of hepcidin expression compared to controls suggesting that endofin is important for the BMP regulation of hepcidin. The importance of endofin in modulating BMP signals was previously shown *in vivo* in *Xenopus* embryos, where endofin regulated BMP-induced mesodermal patterning, as characterised by increased expression of early mesodermal markers Xbra, Xhox3 and Xwnt8. Expression of the FYVE (C753S) mutant showed decreased expression of mesodermal markers despite stimulation with BMPs indicating that endofin is required for BMP signalling^13^.

SMAD anchors are known to mediate TGFβ signalling by interacting and localising SMAD proteins to TGFβ receptors. However, recent findings through co-immunoprecipitation (co-IP) show that they are dispensable for the pathway^28^,^29^. For the first time, we demonstrate an *in situ* interaction of endofin and SARA with SMAD1 and SMAD2/3, respectively. This validates previous reports on the role of SMAD anchors in mediating TGFβ signalling^12^,^13^,^30^,^31^. In addition, we found that endofin interacted similarly to SMAD2/3 suggesting a possible role of endofin in both the BMP and TGFβ signalling pathways. This was previously shown in the human embryonic kidney cell line, HEK293T, and other human hepatic cell lines, Hep3B and HepG2^32^.

To determine the underlying mechanism of hepcidin regulation by endofin, we examined the effects of endofin silencing on SMAD activation. SMAD1/5/8 proteins are receptor-regulated SMADs that transduce BMP signals from membrane receptors to the nucleus and activate the expression of BMP-responsive genes. We observed that the knockdown of endofin caused a decrease in SMAD1/5/8 phosphorylation in both BMP6-treated and untreated control cells. Measurement of relative hepcidin mRNA expression correspondingly showed a down-regulation following endofin knockdown suggesting that endofin mediates SMAD1/5/8 phosphorylation which subsequently influences hepcidin expression. This is similar to previous findings where endofin facilitates expression of BMP-regulated genes through its interaction with different SMADs. Endofin was shown to enhance BMP-SMAD signalling by interacting with unphosphorylated SMAD1 and anchoring SMAD1 to BMP receptors for activation^13^. Endofin also enhances BMP-SMAD signalling by interacting with SMAD4, recruiting SMAD4 to activated SMADs to mediate SMAD complex formation^32^. These SMAD complexes then shuttle to the nucleus to induce transcription of targeted genes.

Deleterious mutations in genes encoding hepcidin or its regulators result in dysregulated production of hepcidin and cause genetic disorders of iron homeostasis. As endofin was able to modulate hepcidin expression, we carried out further studies to determine if endofin was a potential genetic modifier of iron-related disorders. Endofin variants from SNP databases such as HAPMAP, 1000 Genomes Project and NCBI dbSNP were analysed using protein prediction tools; we identified six variants that were potentially deleterious. Characterisation via indirect immunofluorescence showed that the FYVE domain of endofin was important for its localisation to endosomes. Endofin SNP variants with an intact FYVE domain showed endosomal localisation patterns similar to the wild-type and endogenous endofin protein. However, variants containing a premature stop codon or a non-synonymous mutation in the conserved FYVE domain showed a diffused localisation pattern resembling the ER. Localisation of these FYVE domain variants were consistent with a previously reported synthetic mutant FYVE (C753S) that also disrupts the FYVE domain^14^. As the FYVE domain is essential in recruiting SMADs to early endocytic compartments, it could potentially affect the SMAD trafficking function of endofin and disrupt hepcidin transcription^15^. Previous findings have associated a disrupted FYVE domain with delay in membrane trafficking, reduced SMAD activity and attenuation of BMP signalling^13^,^14^,^32^. It is thus possible that endofin may act as a genetic modifier in iron-related disorders such as iron overload caused by *HFE* C282Y, where phenotypic variance is displayed despite homozygous mutations. As such, it would be of interest to assess *in vivo* effects of naturally occurring endofin SNPs with other genetic mutations that cause iron-related disorders. A SNP in endofin has been previously associated with susceptibility to psoriasis, a disease where TGFβ and SMAD signalling have been implicated^33^,^34^. However, endofin SNPs have not been observed in genome wide association studies that have investigated alterations in iron parameters^35^,^36^.

Since endofin is involved in endosomal trafficking, it remains unclear if endofin specifically modulates the BMP-SMAD signalling pathway. Endofin is described in previous studies to be associated with other signalling pathways such as the mitogen-activated protein kinase (MAPK) pathway and the closely-related TGFβ-SMAD signalling pathway^24^,^32^. Endofin is a tyrosine target of EGF signalling in A431 cells^37^. Expression of endofin constructs with a mutated Y515 tyrosine phosphorylation site in HeLa cells enhanced ERK2 phosphorylation, suggesting that endofin plays a role in modulating the MAPK/ERK signalling pathway^24^. In another study, endofin was shown to affect the TGFβ-SMAD signalling pathway by interacting with TGFβ type I receptors^32^. Furthermore, the knockdown of endofin in Hep3B cells attenuated SMAD2-responsive gene expression^32^. However, the study also reported that the influence of endofin on TGFβ-SMAD signalling was specific and did not affect BMP or Wnt signalling. Future work involves further characterising the specific signalling pathways regulated by endofin.

**Figure 6:**
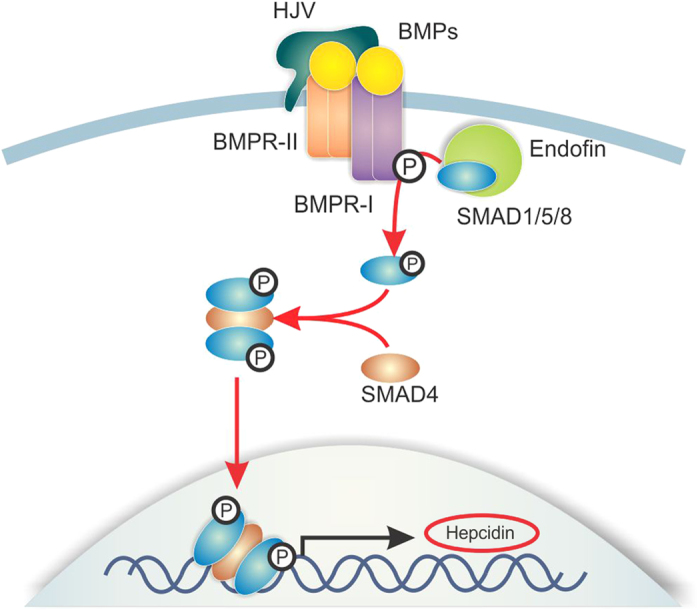
Schematic diagram of hepcidin regulation by endofin through the BMP-SMAD pathway. Upon BMP ligand binding, Type II Receptors phosphorylate Type I Receptors at the glycine-serine domain resulting in its activation, and phosphorylation of the R-SMADs, SMAD1/5/8, which subsequently form heterocomplexes with SMAD4 before translocating to the nucleus to activate the transcription of hepcidin. Endofin, which recruits SMADs to BMP receptors, mediates SMAD1/5/8 phosphorylation and consequently regulates hepcidin expression.

In conclusion, we have identified endofin as an important signalling component required for basal and BMP-induced hepcidin expression. Endofin influences hepcidin expression by regulating SMAD1/5/8 phosphorylation which is central for the transcription of hepcidin (Fig. 6). Identification of the hitherto unrecognised role of endofin further elucidates the molecular network involved in hepcidin regulation which may aid in the management of iron-related disorders.

## Materials and Methods

### Cell Culture

HepG2/C3A hepatoma cells (cat. no. CRL-10741, ATCC, Manassas, VA, USA) were maintained in Minimum Essential Medium (MEM) containing 10% FCS.

### Transfection with DNA and small interfering RNA (siRNA) constructs

#### DNA transfection

C3A cells were grown in 12-well cluster plates (~4 cm^2^) to 80–90% confluence before transfection. pDHA-Neo-Endofin plasmids, described previously^14^, were transfected using Lipofectamine 3000 (Life Technologies, Mulgrave, Australia) according to the manufacturer’s recommendations. Cells were incubated with the DNA-Lipofectamine complexes (100 μl per well) for 48 hours.

#### siRNA transfection

C3A cells were counted and grown to 30–40% confluence in 12-well cluster plates (~4 cm^2^) before transfection with 20 pmol of non-specific or endofin siRNA (GenePharma, Shanghai, China) in complex with Lipofectamine RNAiMAX (Life Technologies). Sequences of the siRNAs used were as follows: non-specific (si-NS) forward 5′-UUCUCCGAACGUGUCACGUTT-3′ and reverse 5′-ACGUGACACGUUCGGAGAATT-3′, endofin (si-*Endofin*) forward 5′-GGGCAAGACUUAGAUUACUTT-3′ and reverse 5′-AGUAAUCUAAGUCUUGCCCTT-3′. Briefly, the siRNAs were diluted in 50 μl Opti-MEM and mixed gently. Subsequently, 2 μl of Lipofectamine RNAiMAX was diluted in 50 μl Opti-MEM and mixed gently. The siRNA-Lipofectamine complexes were mixed gently and incubated at room temperature for 5–10 min. The complex (100 μl) was subsequently added to each well of cells and incubated for 72 hours before harvesting.

### Real-Time PCR analysis of mRNA transcripts

Total RNA was isolated from cells using Trizol reagent (Life Technologies) according to the manufacturer’s recommendations. RNA (1 μg) was reverse transcribed into cDNA using Superscript III (Life Technologies). Reactions were performed on the Viia7 Real Time PCR System (Life Technologies) as described previously^38^. Primer pairs for detecting mRNA transcripts are described in Table 1. All targets were normalised to the geometric means of reference genes, *ACTB* and *HPRT* using 2^−ΔCT^.

### Proximity Ligation Assays (PLA)

The mouse/rabbit red starter Duolink kit (Sigma-Aldrich) was used for this experiment. HepG2/C3A cells were seeded at 10 × 10^4^ cells per well in a 12-well cluster plate and transfected with pDMyc-Neo-Endofin^14^ or pDMyc-Neo-SARA^14^ plasmids as described above. The cells were fixed and permeabilised as described previously^39^. After permeabilisation the cells were incubated in the blocking buffer (provided with the kit) for 1 hr at 37 °C in a humidified chamber. Next, cells were incubated with the primary antibodies diluted in the antibody diluents for 1 hr at room temperature. Primary antibodies used were mouse anti-Myc 9B11 (1:2000; Cell Signaling Technology), rabbit anti-SMAD1 (1:50; Life Technologies), rabbit anti-SMAD2/3 (1:800; Cell Signaling Technology), mouse anti-TfR1 (1:500; Life Technologies) and rabbit anti-TfR1 (1:100; Abcam, Cambridge, UK). Cells were then washed in Buffer A (supplied with the kit) 2 times for 5 min each and incubated with the PLA probes for one hour at 37 °C in a humidified chamber. Cells were washed 2 times for 5 min each in Buffer A before the ligation step at 37 °C for one hour in a humid chamber. Following 2 times of 2 min washes, cells were incubated with the amplification mix for 2 hr at 37 °C in a darkened humidified chamber. Cells were washed with 1 × Buffer B (supplied with the kit) 2 times for 10 minutes followed by a 1 min wash with 0.01 × Buffer B before mounting on cover slips using the mounting media supplied with the kit.

### Western blotting

Cell lysates were homogenised in phosphatase inhibitor lysis buffer and western blotting was carried out as previously described^40^. Blots were incubated with antibodies: anti-endofin^14^ (1:30000), anti-phospho-SMAD1/5/8 (1:3000; Cell Signaling Technology, Danvers MA, USA), anti-HA 12CA5 hybridoma supernatant (1:2000; ATCC, Manassas, VA, USA) and anti-actin (1:20000; Sigma-Aldrich, Missouri, USA). Bands developed on film were scanned using Scanmaker 9800 XL plus (Microtek International Inc. Hsinchu, Taiwan) and subsequently quantified by densitometry using the GeneGenius Imaging System (Syngene, Cambridge, UK).

### Mutagenesis

Endofin variants were identified through SNP database searches (http://www.ncbi.nlm.nih.gov/projects/SNP) and were analysed using Sorting Intolerant From Tolerant (SIFT; http://sift.jcvi.org/) and Polymorphism Phenotyping v2 (PolyPhen-2; http://genetics.bwh.harvard.edu/pph2/) programs. Six variants (rs13157990, rs61741923, rs16877836, rs35915800, rs34397330 and rs35033083) with potentially deleterious mutations, as predicted by these programs, were selected for mutagenesis. All primers designed to introduce the site-directed mutation were synthesised by Integrated DNA Technologies (IDT, Coralville, Iowa). Mutations were introduced into HA-tagged endofin constructs in pDHA^14^ with the primers listed in Table 2. Mutagenesis was then carried out according to Scott *et al.*^41^ with changes as noted: a PCR mixture containing 75 ng template plasmid DNA, 2 μM of each oligonucleotide, 2 mM dNTPs, 50 U KOD DNA polymerase (Merck Chemicals, Victoria, Australia) and polymerase buffer in a total volume of 50 μl. PCR was performed under the following conditions; denaturation at 95 °C for 2 min, followed by 18 cycles of denaturation at 95 °C for 20 seconds, annealing at 58 °C for 10 seconds and extension at 68 °C for 5 minutes. The endofin insert was sequenced to ensure that the generated mutation was present and no other unintentional sequence changes had been inserted during the mutagenesis procedure.

### Indirect immunofluorescence microscopy

Cultured cells were grown on glass coverslips overnight and were prepared for immunofluorescence as described previously^42^. Primary antibodies used to assess expression were rabbit anti-endofin^14^ (1:500), rabbit anti-HA H6908 (1:100; Sigma-Aldrich), mouse anti-EEA1 (1:400; BD Transduction Labs, San Jose, CA, USA) and rabbit anti-ERp57^43^ (1:600; a gift from Prof. Stephen High, University of Manchester, Manchester, UK). Cells were mounted onto glass slides using ProLong Gold anti-fade with 4′, 6-diamidino-2-phenylindole dihydrochloride (DAPI) (Molecular Probes, Life Technologies, Mulgrave, Australia). Fluorescent images were viewed and captured using a Zeiss LSM 780 NLO inverted confocal microscope (Carl Zeiss, Oberkochen, Germany).

### Statistical analysis

Statistics were generated using GraphPad Prism software v.6.0 (GraphPad Software, San Diego CA, USA). Results were expressed as the mean ± standard error. Variables were compared between groups using two way analysis of variance (2-way ANOVA). Post-hoc analysis was performed to compare the differences between groups using Tukey’s multiple comparison test. Statistical significance is indicated as follows: *indicates a p-value of < 0.05, **p < 0.01, ***p < 0.001. Graphs were prepared using Graphpad Prism 6.

## Additional Information

**How to cite this article**: Goh, J. B. *et al.* Endofin, a novel BMP-SMAD regulator of the iron-regulatory hormone, hepcidin. *Sci. Rep.* **5**, 13986; doi: 10.1038/srep13986 (2015).

## Figures and Tables

**Table 1:** Sequence of primer pairs used for qPCR.

Gene	Primer	Sequence
***ACTB*** (***β-ACTIN***)	F	CAGGCACCAGGGCGTG
	R	GCCCACATAGGAATCCTTCTGA
***HPRT1***	F	GAAAGGGTGTTTATTCCTCAT
	R	CCCATCTCCTTCATCACAT
***HAMP*** **(Hepcidin)**	F	CCACAACAGACGGGACAAC
	R	AAAATGCAGATGGGGAAGTG
***ID1***	F	TGGAGCTGAACTCGGAATCCG
	R	GACACAAGATGCGATCGTCCG
***SMAD7***	F	TCACCTTAGCCGACTCTGCG
	R	GTTTCAGCGGAGGAAGGCAC
***ZFYVE9*** **(SARA)**	F	CTGTGCTTCCTGCTGTAGCCTGAAA
	R	TTAGGGCTCTGGCTTGAGGCACT
***ZFYVE16*** **(Endofin)**	F	ATGGCTTGTAGTGCTGCGCTGT
	R	AGGCAGAAGTTGGCCTTCAGATCC

**Table 2:** Oligonucleotide primers used for site-directed mutagenesis.

**SNP ID**	**Primer**	**Sequence**
**rs13157990 R766G**	F	TTACTTTTACCAAACGG**G**GACACCATTGCCGAGCA
	R	TGCTCGGCAATGGTGTC**C**CCGTTTGGTAAAAGTAA
**rs61741923 E1431Q**	F	GAGAAGATTGTAAAATGTACC**C**AGGTGTTCTACTTTCTAAAGG
	R	CCTTTAGAAAGTAGAACACCT**G**GGTACATTTTACAATCTTCTC
**rs16877836 T1519N**	F	TGTGATCCATGGTGGGA**A**CTCCAACTCTAGTTTAC
	R	GTAAACTAGAGTTGGAG**T**TCCCACCATGGATCACA
rs35915800 K325Qfs*4	F	ATTCAAATTCAAGAGATGAAAA**T**TTTCAAATTACCTGACTTTTCC
	R	GGAAAAGTCAGGTAATTTGAAA**A**TTTTCATCTCTTGAATTTGAAT
rs34397330 N729Kfs*19	F	CAAATGAAGATTCTGTACCTG**A**AAAACACTTGCAAAGAAGGCT
	R	AGCCTTCTTTGCAAGTGTTTT**T**CAGGTACAGAATCTTCATTTG
rs35033083 T928Yfs*8	F	AGTGGAAAAGCCAAACAATGAG**T**ACAGGAGATATTACAAGAAATG
	R	CATTTCTTGTAATATCTCCTGT**A**CTCATTGTTTGGCTTTTCCACT

Mutant bases are indicated in bold.
